# Influence of organ donor attributes and preparation characteristics on the dynamics of insulin secretion in isolated human islets

**DOI:** 10.14814/phy2.13646

**Published:** 2018-03-13

**Authors:** Jean‐Claude Henquin

**Affiliations:** ^1^ Unit of Endocrinology and Metabolism Faculty of Medicine University of Louvain Brussels Belgium

**Keywords:** Human islets, insulin secretion, islet donor age, islet donor BMI, islet size

## Abstract

In vitro studies of human pancreatic islets are critical for understanding normal insulin secretion and its perturbations in diabetic *β*‐cells, but the influence of islet preparation characteristics and organ donor attributes in such experiments is poorly documented. Preparations from normal donors were tested with a standardized protocol evaluating dynamic insulin secretion induced by glucose, tolbutamide, and cAMP (forskolin). Secretion rates, normalized to insulin content (fractional insulin secretion), were analyzed as a function of preparation and donor characteristics. Low purity (25–45%) of the preparation (*n* = 8) blunted the first phase of insulin secretion induced by glucose or tolbutamide and increased basal secretion, resulting in threefold lower stimulation index than in more pure (55–95%) preparations (*n* = 43). In these more pure preparations, cold ischemia time (1–13 h) before pancreas digestion did not impact insulin secretion. Islet size (estimated by the islet size index) did not influence the dynamics of secretion, but fractional insulin secretion rates were greater in large than small islets, and positively correlated with islet size. Age of the donors (20–68 years) had no influence on islet size and insulin content or on dynamics and amplitude of insulin secretion, which were also similar in islets from male and female donors. In contrast, islet size and islet insulin content (normalized for size), and basal or stimulated insulin secretion positively correlated with Body‐Mass Index (19–33). These results contradict previous reports on the impact of donor age and islet size and point to possible confounding effects of donor BMI in insulin secretion studies with isolated human islets.

## Introduction

Insulin, secreted by *β*‐cells of pancreatic islets, is indispensable to ensure glucose homeostasis. In all types of diabetes, hyperglycemia results from an absolute or relative insufficiency of insulin secretion, whereas excessive secretion causes life‐threatening hypoglycemia. It is not surprising therefore that the mechanisms regulating insulin secretion have been extensively investigated. Although in vivo studies have long been carried out in human subjects, our detailed knowledge of the mechanisms controlling *β*‐cell secretory function largely rests on the in vitro use of rodent islets. Yet, in parallel with the development of clinical programs of islet isolation for transplantation, human islets have progressively become available for experimental research in various areas (Kaddis et al. 2009; Nano et al. 2015). Although the control of insulin secretion is basically similar in human and mouse *β*‐cells (Henquin et al. 2017a; Rorsman and Ashcroft 2018), its glucose dependency is strikingly shifted to the left in human *β*‐cells (Henquin et al. 2006; Doliba et al. 2012). Recent studies have evidenced further species‐dependent peculiarities in various facets of stimulus‐secretion coupling: nutrient metabolism (MacDonald et al. 2011; Doliba et al. 2012), biophysical events generating electrical activity and leading to a rise in *β*‐cell cytosolic Ca^2+^ concentration (Fridlyand et al. 2013; Skelin Klemen et al. 2017; Rorsman and Ashcroft 2018), and neuro‐hormonal regulation (Amisten et al. 2017). The importance of in vitro studies of human islets is now widely recognized, and the preparation is likely to become the gold standard in a near future.

However, switching from relatively homogeneous rodent models to human islets is not without pitfalls. Although donor and organ variables impacting the islet isolation outcome, in particular yield and probability of post‐transplantation functioning, have been extensively investigated (Kaddis et al. 2010; Balamurugan et al. 2014; Hilling et al. 2014), relatively little attention has been paid to donor and preparation characteristics that might influence insulin‐secreting properties of normal human islets in vitro (Lyon et al. 2016). In previous studies, we characterized the control of insulin secretion by nutrients (Henquin et al. 2006) or pharmacological agents (Henquin et al. 2017a), and the biphasic response to glucose stimulation (Henquin et al. 2015) in perifused human islets. The quality of the preparations was ascertained using a standardized stimulation protocol testing the two phases of insulin secretion induced by glucose and tolbutamide, and amplification of the response by cAMP. In this report, the results of these control experiments were re‐analyzed to assess how features of the preparation (purity, culture duration, islet size, and cold ischemia time) and donor attributes (sex, age, and BMI) influence insulin secretion in vitro.

## Methods

Human pancreatic islets were isolated from nondiabetic, adult, organ donors in transplantation units of the Medical Faculties of the University of Louvain in Brussels (Dufrane et al. 2005) and the University of Lille (Kerr‐Conte et al. 2010). Approval of the experimental use of these islets was granted by ethical committees of both institutions and consent was given by the donor's family.

**Figure 1:**
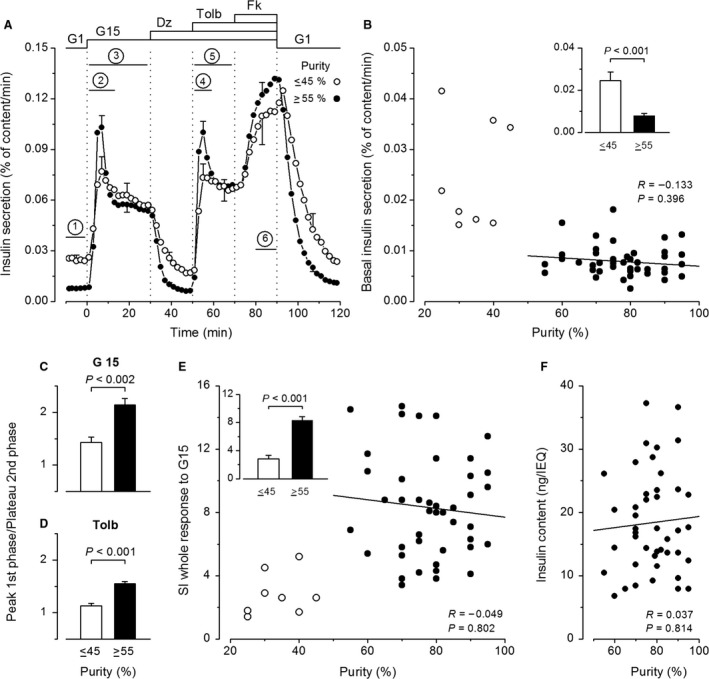
Influence of the purity of human islet preparations on insulin secretion. (A) Dynamics of insulin secretion in preparations with purities ≤45% or ≥55% (*n* = 8 and 43, respectively). Experiments started with a 60‐min stabilization period, of which only the last 10 min are shown. Between 0 and 90 min, the concentration of glucose was increased from 1 (G1) to 15 mmol/L (G15). Diazoxide (Dz; 100 *μ*mol/L), tolbutamide (Tolb; 100 *μ*mol/L), and forskolin (Fk; 1 *μ*mol/L) were added and withdrawn as indicated. Bars labeled 1–6 show time periods over which insulin secretion rates were averaged for subsequent analyses as described in Results. (B) Basal insulin secretion rate in G1. (C–D) Ratio of secretion rates at peak of first phase and plateau of second phase of responses to G15 (C) and Tolb (D). (E) Stimulation index (SI) for the whole insulin response to G15. (F) Islet insulin content normalized to islet equivalents (IEQ). A, C and D, and insets in B and E compare means ± SE for preparations with low and high purity. *P* values were calculated by Mann–Whitney test. B, E and F show individual values as a function of the purity of each islet preparation. Correlation coefficients were calculated by the test of Spearman for the group of high purity only.

The dynamics of insulin secretion by these islets was studied using a perifusion technique that has been described in detail (Henquin et al. 2006, 2015). After isolation, islets were cultured at 37° for 45.8 ± 2.2 h (range 22–79). A defined portion of the preparation was then distributed into eight parallel perifusion chambers, in which islets were subjected to different tests, including a standardized protocol of quality control (Henquin et al. 2015). A similar volume of tissue was transferred into the eight chambers, but the exact number of islets was not determined for each chamber. At the end of experiments, the tissue was recovered from the chambers for insulin extraction in acid‐ethanol (Detimary et al. 1996). Secreted insulin was measured in effluent fractions and expressed as a function of the insulin content of the islets perifused in the same chamber. Reported insulin secretion rates thus correspond to fractional secretion rates (percentage of islet insulin content secreted per minute), which are independent of differences in islet numbers between experiments (Henquin et al. 2006, 2015). Fifty‐one preparations were studied with the same test protocol designed to evaluate the two phases of insulin secretion induced by glucose and tolbutamide, and the amplification of the response by cAMP (Fig. 1A). The results of some of these control experiments were included in previous publications (Henquin et al. 2006, 2015).

For each studied preparation, the total number of received islets was determined, in parallel with assessment of islet viability (trypan blue exclusion) and purity (dithizone staining). The average islet size was estimated as the “islet size index,” by computing the ratio of “islet equivalents” (islets with a theoretical diameter of 150 *μ*m) to the total number of islets (Suszynski et al. 2014). An islet size index of 1.0 would mean that islets in the preparation have an average volume similar to that of a spherical islet with a diameter of 150 *μ*m. The average islet insulin content was estimated from the sum of insulin contents measured in the eight perifusion chambers divided by the total number of islets distributed into these chambers, and was normalized per islet equivalent.

Results are presented as scatter plots of individual values or means ± SE. The impact of donor and preparation characteristics was evaluated by linear regression and after data stratification into two categories with median cutoffs. Because values were not always normally distributed, the statistical significance of differences between two compared groups was assessed by the nonparametric Mann–Whitney test, and correlations between variables were assessed by the test of Spearman.

## Results

### The test protocol

Figure 1A shows the protocol that was used to characterize the dynamics of insulin secretion in 51 islet preparations, and delineates the six periods over which average secretion rates were computed for comparisons between groups. Experiments started with a 60‐min period of perifusion with a medium containing 1 mmol/L glucose (G1) to establish baseline insulin secretion, which was quantified between −10 and 0 min (period 1). Islets were then stimulated with G15 alone for 30 min, which resulted in a typical biphasic increase in insulin secretion (Henquin et al. 2015). This response was quantified during the first phase (period 2: between 2 and 12 min), and the whole stimulation (period 3). Glucose‐induced insulin secretion was then inhibited by opening ATP‐sensitive K^+^ channels with 100 *μ*mol/L diazoxide between 30 and 50 min, and this inhibition was reversed by closing the channels with 100 *μ*mol/L tolbutamide. The biphasic response to tolbutamide was quantified over the first phase (period 4: between 52 and 60 min), and the whole stimulation (period 5). At 70 min, 1 *μ*mol/L forskolin was added to increase islet cAMP concentrations and amplify insulin secretion. The response was quantified between 80 and 90 min (period 6). At 90 min, all test agents were withdrawn and islets were again perifused with G1 alone, which resulted in a return of secretion rates to basal levels (Fig. 1A).

### The influence of islet purity

Among the 51 islet preparations, a subgroup of eight preparations was characterized by a low purity (33.8 ± 2.6%; range 25–45%), as compared with the other 43 preparations that were >55% pure (78.0 ± 1.7%). Islet viability was also lower in the group with low purity (85.6 ± 3.1%) than in the group with high purity (92.4 ± 0.6%, *P* < 0.05). The dynamics of insulin secretion by the two groups is compared in Figure 1A. The general pattern was similar, but low‐purity preparations showed a threefold elevation of basal secretion in G1 (Fig. 1A and inset of 1B). In only two preparations with purities >55% did baseline secretion rates overlap those measured in low‐purity preparations (Fig. 1B). The second feature of low‐purity preparations was a blunted first phase response to both G15 and tolbutamide, whereas second phases were similar (Fig. 1A). Although average rates of insulin secretion during first phases were not statistically different between the two groups (*P* = 0.08 and *P *= 0.09), the negative impact of low purity was demonstrated by lower ratios between secretion rates at the peak of first phases and plateau of second phases (Fig. 1C and D). Because of high basal secretion rates, the stimulation index (ratio of secretion in G15/G1) was lower in low‐purity than in most other islet preparations (Fig. 1E), with an average 3.3‐fold difference for the first phase and a 2.9‐fold difference for the whole response (Fig. 1E, inset). To permit comparisons between preparations, the islet insulin content was normalized to islet size (islet equivalent). Normalized insulin content of low‐purity islets ranged from 6.3 to 27.2 ng/islet equivalent, values within those measured in more pure preparations (Fig. 1F).

In the group of 43 islet preparations with purities between 55 and 95%, no correlation was found between purity and basal insulin secretion in G1 (Fig. 1B), amplitude of the responses to G15, tolbutamide or forskolin (Table 1), or stimulation index of G15 (Fig. 1E). Normalized islet insulin content was also independent of purity (Fig. 1F). To avoid the confounding influence of low purity, all subsequent analyses of insulin secretion characteristics were restricted to the group of 43 preparations with a purity of at least 55%.

**Table 1:** Correlations between islet preparation characteristics or donor attributes and insulin secretion in isolated human islets

	Baseline	First G15	Whole G15	First Tolb	Whole Tolb	Forskolin	SI First G15	SI Whole G15
**Islet purity**	−0.133 *0.396*	−0.169 *0.279*	−0.176 *0.260*	−0.257 *0.096*	−0.281 *0.068*	−0.184 *0.243*	−0.011 *0.901*	−0.049 *0.802*
**Islet size index**	0.398 ***0.008***	0.433 ***0.004***	0.498 ***0.001***	0.451 ***0.002***	0.438 ***0.003***	0.454 ***0.003***	0.069 *0.661*	0.107 *0.494*
**Cold ischemia time**	0.380 ***0.013***	0.030 *0.850*	0.114 *0.473*	0.272 *0.082*	0.263 *0.092*	0.251 *0.109*	−0.220 *0.162*	−0.170 *0.281*
**Donor age**	−0.115 *0.465*	−0.281 *0.069*	−0.218 *0.160*	−0.301 ***0.050***	−0.311 ***0.042***	−0.248 *0.114*	−0.151 *0.332*	−0.070 *0.656*
**Donor BMI**	0.190 *0.222*	0.380 ***0.012***	0.432 ***0.004***	0.334 ***0.028***	0.337 ***0.027***	0.389 ***0.011***	0.228 *0.142*	0.280 *0.069*

Correlation coefficients *R* were calculated by the test of Spearman and are presented with *P* value in italics.

Bold characters are used to highlight *P* values (0.05 or lower) indicating statistical significance of the correlation coefficient.

The six periods over which insulin secretion rates were averaged are defined in Figure 1.

SI, Stimulation Index was calculated for the first phase and the whole response to G15.

Extending culture time beyond 4 days was previously found to decrease insulin content and stimulation index in human islets (Lyon et al. 2016). Virtually all (40/43) of our experiments were performed between 1 and 3 days of culture. There was no correlation between these relatively short culture periods and either islet insulin content (*P* = 0.94) or the stimulation index of G15 (*P* = 0.87).

### The influence of islet size

**Figure 2:**
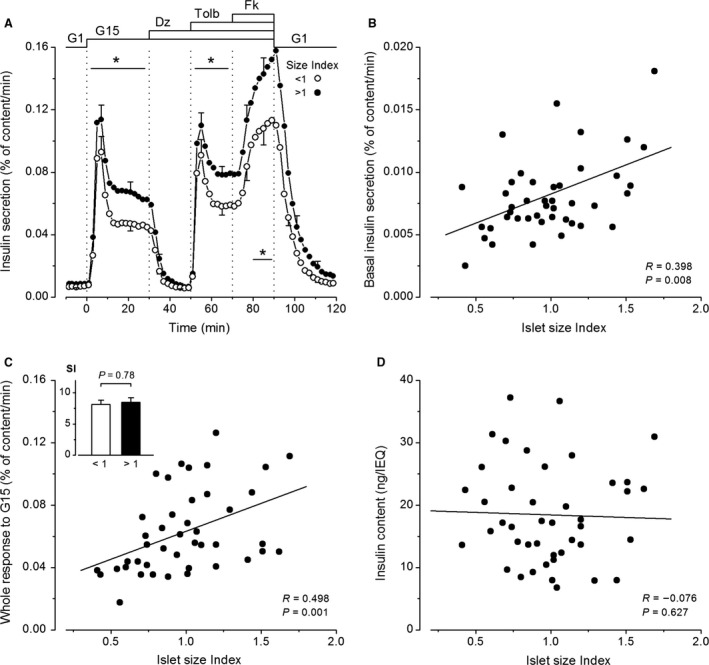
Influence of islet size (expressed as islet size index of the preparation) on insulin secretion by isolated human islets. (A) Dynamics of insulin secretion in islet preparations with a size index lower or higher than 1.0. Values are means ± SE for 21 and 22 islet preparations. Significant differences between the two groups during reference periods are denoted by *(*P* < 0.05) (Mann–Whitney test). (B–D) Basal insulin secretion rate in G1 (B), whole insulin response to G15 (C) and normalized islet insulin content (D), as a function of the islet size index. Correlation coefficients were calculated by the test of Spearman. (C) The inset compares stimulation index (SI) for whole insulin responses to G15 (means ± SE) in islet preparations with an islet size index below or above 1.

The average islet size in each preparation was estimated as the islet size index. Among the 43 preparations, the size index ranged from 0.41 to 1.69, with a mean of 0.99 ± 0.05 and a median of 0.98. As shown in Figure 2A, the dynamics of insulin secretion was similar in 22 islet preparations with a size index lower than 1.0 (mean of 0.74 ± 0.03) and 21 preparations with a size index greater than 1.0 (mean of 1.25 ± 0.05). Secretion rates were higher in larger than smaller islets, the difference (25–30%) being significant (*P* < 0.05) except during the baseline period and first phases of the responses to glucose or tolbutamide, where *P* was between 0.10 and 0.05 (Fig. 2A). However, insulin secretion rates during baseline and all phases of stimulation increased with the islet size index (Fig. 2B and C) (Table 1). Because both basal and stimulated insulin secretion augmented with islet size, the stimulation index did not (Table 1) (Fig. 2C, inset). In the whole group, normalized insulin content varied 5.5‐fold (from 6.8 to 37.2 ng per islet equivalent), averaged 18.4 ± 1.2 ng per islet equivalent, and did not correlate with the islet size index of the preparation (Fig. 2D).

### The influence of cold ischemia time

**Figure 3:**
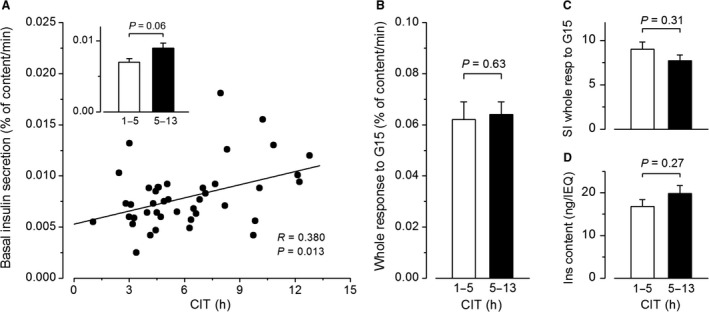
Influence of cold ischemia time (CIT) on insulin secretion by isolated human islets. (A) Basal insulin secretion rates in G1 as a function of CIT. The correlation coefficient was calculated by the test of Spearman. (B) Whole insulin response to G15. (C) Stimulation index (SI) for the whole response to G15. (D) Normalized islet insulin content. The inset in A, and B–D compare means ± SE for 20 preparations with short (1–5 h) and 22 preparations with longer (5–13 h) CIT (Mann–Whitney test).

Cold ischemia time (CIT) is the interval between pancreas cooling with a preservation solution at harvesting from the donor and initiation of the islet isolation procedure. Increasing CIT negatively affects the yield of human islet isolations (Hilling et al. 2014). Among our preparations, CIT ranged from 1.1 to 12.7 h, with a mean of 6.1 ± 0.5 h and a median of 5.1 h. Basal insulin secretion rates in G1 increased with CIT (Fig. 3A), but the difference between preparations with CIT below and above 5 h was modest and not quite statistically significant (Fig. 3A, inset). CIT did not affect the whole insulin response to G15 (Fig. 3B) or any phase of stimulated insulin secretion (Table 1), and its influence on basal secretion was too small to impact the stimulation index of G15 (Fig. 3C). Normalized islet insulin content was also unaffected by CIT (Fig. 3D). Overall, the results show that CIT between 1 and 13 h has no impact on insulin secretion by isolated human islets.

### The influence of donor sex

**Figure 4:**
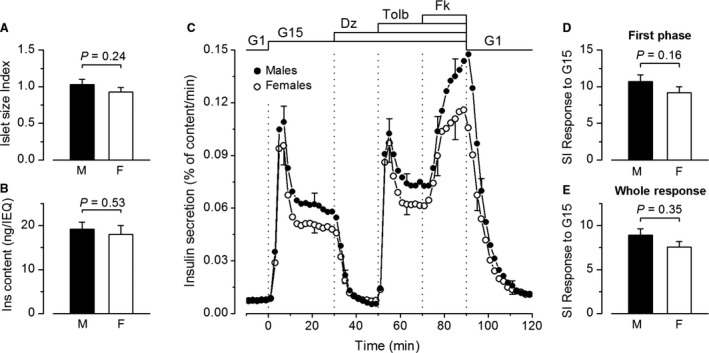
Influence of donor sex on insulin secretion by isolated human islets. (A) Islet size index. (B) Normalized islet insulin content. (C) Dynamics of insulin secretion. (D–E) Stimulation index (SI) for the first phase and the whole response to G15. Values are means ± SE for 24 islet preparations from male donors and 19 preparations from female donors. All comparisons by Mann–Whitney test.

Among the 43 donors, 24 were males and 19 females. Males were younger than females (43.0 ± 2.7 years vs. 52.1 ± 1.9 years; *P *= 0.015), whereas BMI was not different between the two groups (26.1 ± 0.7 vs. 24.8 ± 0.8; *P* = 0.136). Islets isolated from male or female donors had similar sizes (Fig. 4A) and normalized insulin contents (Fig. 4B). The dynamics of insulin secretion was virtually identical in islets of each sex and the trend toward higher secretion rates in male than female islets (~15%) was not significant (*P* > 0.275) during any of the phases of stimulation (Fig. 4C). The stimulation index of G15 was similar in islets from male and female donors during both the first phase (Fig. 4D) and the whole response to G15 (Fig. 4E).

### The influence of donor age

**Figure 5:**
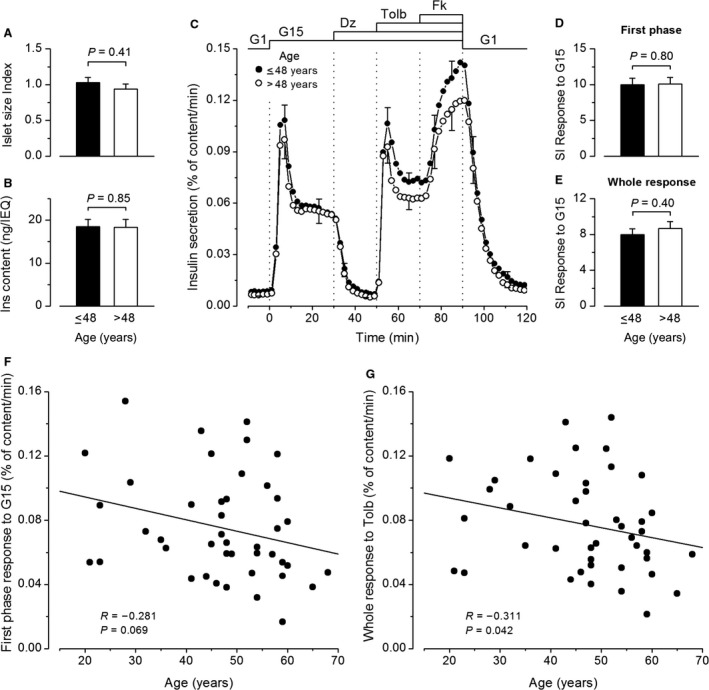
Influence of donor age on insulin secretion by isolated human islets. (A) Islet size index. (B) Normalized islet insulin content. (C) Dynamics of insulin secretion. (D–E) Stimulation index (SI) for the first phase and the whole response to G15. Values are means ± SE for 23 islet preparations from donors aged ≤48 years and 20 preparations from donors aged >48 years. All comparisons by Mann–Whitney test. (F–G) First phase insulin response to G15 (F) and whole insulin response to tolbutamide (G) as a function of donor age. Correlation coefficients were calculated by the test of Spearman.

The age of the 43 donors ranged from 20 to 68 years, with a mean of 47.0 ± 1.8 years and a median of 48.0 years. Neither islet size index nor normalized insulin content of the islets differed between preparations from donors younger or older than 48 years (Fig. 5A and B). The dynamics and the amplitude of insulin secretion were similar in islets from the two age groups (Fig. 5C). Only indices suggestive of minor deterioration of insulin secretion with aging could be found through correlations. The first phase of the response to G15 tended to decrease slightly (not quite significantly) with increasing age (Fig. 5F), but the whole response was unaffected (Table 1). The insulin response to tolbutamide also marginally decreased with aging (Fig. 5G) (Table 1). There was, however, no difference in stimulation index of G15 between islets from donors below and above 48 years (Fig. 5D and E). Overall, the donor age has little impact on insulin secretion when studied in vitro with isolated islets.

### The influence of donor BMI

**Figure 6:**
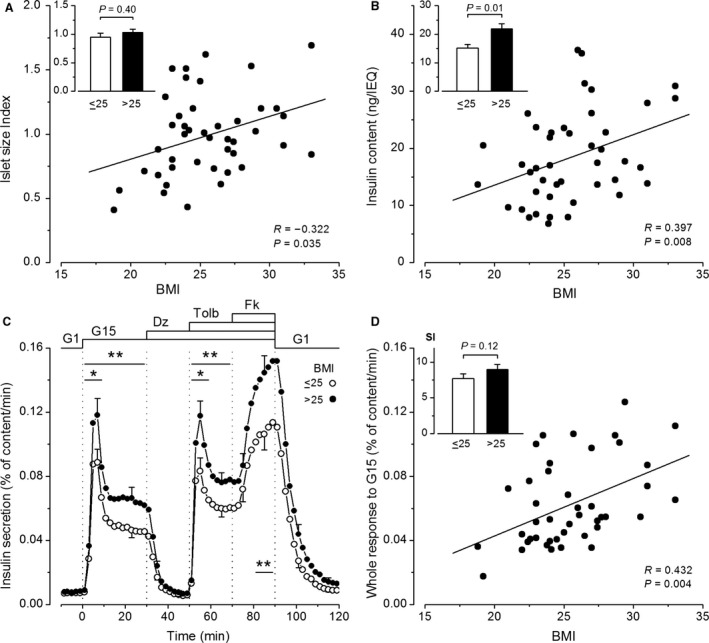
Influence of donor BMI on insulin secretion by isolated human islets. (A) Islet size index. (B) Normalized islet insulin content. (C) Dynamics of insulin secretion. (D) Whole insulin response to G15 and stimulation index (SI) for this response (inset). A, B and D show individual values as a function of donor BMI. Correlation coefficients were calculated by the test of Spearman. C and insets in A, B, and D compare means ± SE for preparations from 22 donors with BMI ≤25 and 21 donors with BMI >25. All comparisons by Mann–Whitney test. In C, significant differences during reference periods are denoted by *(*P* < 0.05) and **(*P *< 0.02).

The BMI of the 43 donors ranged from 18.8 to 33.0, with a mean of 25.5 ± 0.5 and a median of 25.0. The islet size index of the preparations correlated positively with BMI of the donor (Fig. 6A), but mean islet sizes were not statistically different between groups of BMI below and above 25 (Fig. 6A, inset). Normalized insulin content of islets also increased with BMI of the donor (Fig. 6B), and this resulted in a 1.4‐fold difference between groups of lower and higher BMI (Fig. 6B, inset). The dynamics of insulin secretion was similar in islets from the two BMI groups, but the amplitude of responses was greater in islets from donors with a higher BMI (Fig. 6C). With the exception of baseline in G1, all phases of the secretory response were positively correlated with BMI of the preparation donor (Table 1). This is illustrated for the whole response to glucose in Figure 6D. There was, however, no significant influence of BMI on the stimulation index of G15 (Table 1) (Fig. 6D, inset).

## Discussion

For proper interpretation of the above analyses and their comparison with previous reports, some methodological aspects must be borne in mind. In other studies, islets were handpicked and thus inevitably selected before functional evaluation, whereas unselected portions of the islet preparations (several hundreds of islets) were used in our experiments. This approach is expected to provide secretion rates that are representative of the whole islet population in each donor (Henquin et al. 2015). Average islet size was also estimated for the whole preparation (islet size index) and used to normalize the insulin content per islet equivalent (which corresponds to a concentration). The mean value of 18.4 ng insulin per islet equivalent is very close to that obtained by dividing the insulin content of whole autopsy pancreases (Henquin et al. 2017b) by the number of islet equivalents determined by quantitative morphology (Olehnik et al. 2017) (11.3 mg: 600,000 = 18.8 ng). Insulin secretion was expressed as a fractional rate (percentage of content per min), which means that observed differences in secretion are not due to differences between insulin stores in islets but reflect secretion of a greater or smaller proportion of these stores. Finally, whereas most other studies used static islet incubations and a single stimulus, high versus low glucose, to evaluate the impact of islet characteristics and donor attributes on insulin secretion, we monitored the dynamics of secretion in response to several stimuli. Some of our findings agree with previous conclusions, but others do not or are unprecedented, as discussed in the following sections.

### The influence of islet purity

Preparations of digested human pancreas are routinely purified to increase viability of the islets and minimize the mass of nonendocrine tissue infused to receivers (Nano et al. 2005). Surprisingly, how final purity of the preparation influences the characteristics of insulin secretion by isolated islets has not previously been investigated in detail, probably because islets are generally handpicked before study. Using samples of whole preparations, we observed alterations of insulin secretion (high baseline and blunted first phase with lower stimulation index) only in preparations with a purity of 45% or lower. The lesser viability of these islets may have contributed to these alterations, in particular to baseline elevation. In contrast, variations in the preparation purity between 55 and 95% did not induce artefactual variations in insulin secretion, at least within 3 days of islet isolation. Nevertheless, we elected to use only preparations with at least 70% purity in recent studies (Henquin et al. 2015, 2017a). Further purification by islet handpicking would obviously be necessary for metabolic and gene expression analyses.

### The Influence of cold ischemia time

Although it is widely accepted that a long CIT decreases yield and quality of human islets used in transplantation programs (Hilling et al. 2014), the influence of CIT on functional properties of islets used in experimental in vitro studies has received little attention. A recent study (Lyon et al. 2016) reported a negative correlation between islet insulin content and CIT (1–24 h), which was not observed here probably because of shorter CIT (1–13 h) in our preparations. In both studies, glucose‐induced insulin secretion and the stimulation index were independent of CIT. Within the range accepted by most transplantation centers (up to 12 h), CIT therefore has virtually no negative impact on in vitro insulin secretion by human islets.

### The influence of islet size

In our experiments, the dynamics of insulin secretion in response to glucose, tolbutamide, and forskolin was not influenced by islet size, but basal and stimulated secretion rates positively correlated with the islet size index of the preparation. A higher percentage of the insulin content was secreted by islet populations with a larger average size index. These findings are at odds with the common idea that small islets function better than big ones. The discrepancy has several methodological explanations. First, I compared fractional insulin secretion rates in whole preparations containing islets with different average sizes, whereas others selected small and large islets by handpicking in each preparation. Lehmann et al. (2007) observed that, after correction for size (normalization per islet equivalent), both basal and glucose‐stimulated insulin secretion rates were twofold greater in small than big islets, with no difference in stimulation index. Similar conclusions were reached in three other studies that also normalized secretion per islet equivalent (Fujita et al. 2011; Farhat et al. 2013; Ramachandran et al. 2015). In one of these, however, the impact of islet size on islet function was much lesser after normalization of insulin secretion to cell number, an attenuation that was attributed to overestimation of the size of very large islets by the islet equivalent method (Ramachandran et al. 2015). Furthermore, no functional superiority of small islets was found when in vitro insulin secretion was expressed per islet DNA (Steffen et al. 2011).

A second cause of discrepancy is that other studies usually compared islets below and above 150 *μ*m in diameter (up to 400 *μ*m) without providing information on mean sizes in the two categories. From data presented in the study of Lehmann et al. (2007), one can recalculate an islet size index of ~0.55 and ~3.2 for the groups of small and large islets (ratio of volumes = 5.8‐fold). These figures contrast with islet size index of 0.74 and 1.25 in this study (ratio of volumes = 1.7‐fold). Big islets selected by other investigators were thus considerably larger than ours and correspond to a minority of the islets present in a normal pancreas. I therefore conclude that, in vitro, small islets are not functionally better than large islets, except perhaps than the few very big ones. Admittedly, the situation may be quite different for transplanted islets, the survival and functioning of which critically depend on size‐limited oxygen supply (Lehmann et al. 2007; Suszynski et al. 2014).

### The influence of donor sex

Current in vitro studies of human islets do not take the donors' sex into consideration although possible differences have not been formally excluded. Using incubations in low versus high glucose, one study found that the stimulation index was marginally greater in islets from females than males (Hall et al. 2014), whereas the opposite result was found in another study (Lyon et al. 2016). In our series, no islet characteristic or response to any secretagogue was significantly different between male and female islets. Notably, our comparison is confounded by neither BMI, that was similar in the two groups, nor age that had no impact on secretion. Overall, one can conclude that male and female isolated human islets are functionally similar.

### The influence of donor age

As glucose homeostasis progressively deteriorates in aging subjects (Chang and Halter 2003), the potential influence of age on human islet function has been investigated in a number of in vitro studies. Published results are somewhat contradictory partly because of differences in experimental approaches and modes of data expression. Some studies, based on incubations of handpicked islets in low and high glucose, reported that the stimulation index is unchanged in islets from older donors (Street et al. 2004; Niclauss et al. 2011). Others reported a decrease (Ihm et al. 2006; Gregg et al. 2016; Lyon et al. 2016), caused by either elevation of basal secretion (Ihm et al. 2006) or diminution of the stimulation by glucose (Gregg et al. 2016). No impact of age was observed in studies using islet perifusions (Lakey et al. 1996; Almaça et al. 2014). In the most recent report (Westacott et al. 2017), glucose‐induced insulin secretion decreased with donor age (though without impact on stimulation index) when islets were studied in static incubations. In contrast, the influence of age was minimal in perifused islets and differed according to the duration of glucose stimulation: a slower return to basal secretion on cessation of short (9‐min) stimulation and a smaller ratio of first to second phase during 30‐min stimulations (Westacott et al. 2017).

In agreement with others (Gregg et al. 2016; Westacott et al. 2017), neither size nor insulin content of isolated islets was influenced by donor age in the present study. Only trends toward smaller insulin responses to glucose and tolbutamide were disclosed in islets of older donors, without any alteration in the dynamics of insulin secretion changes. Overall, our results and several other studies indicate that aging has little negative impact on intrinsic *β*‐cell function as studied in vitro. In vivo, absolute glucose‐induced insulin secretion does not decrease with aging, but becomes insufficient to compensate for the increasing insulin resistance (Gumbiner et al. 1989; Chang and Halter 2003; Utzschneider et al. 2004; Ohn et al. 2016). The relative secretory deficit present in vivo cannot be detected during in vitro experiments. Extrinsic factors, such as vascularization of the endocrine pancreas (Almaça et al. 2014), are likely to influence in vivo insulin secretion in aged subjects. From a practical point of view, the present analysis suggests that age (between 20 and 68 years) is not a confounding parameter in experimental studies of islet secretory function. However, owing to discrepancies between this and some other studies (Gregg et al. 2016; Lyon et al. 2016; Westacott et al. 2017), the issue is not completely settled and deserves attention by other investigators.

### The influence of donor BMI

Both basal and stimulated insulin secretion is increased in normoglycemic obese subjects (Polonsky et al. 1988; Ferrannini et al. 2004). In previous studies using islet incubations, donor BMI (from 20 to more than 40) influenced neither insulin secretion in low and high glucose nor the stimulation index (Matsumoto et al. 2004; Gregg et al. 2016; Lyon et al. 2016). The average size of isolated islets was found to increase with BMI (Matsumoto et al. 2004; Wang et al. 2013; Lyon et al. 2016), but no difference in insulin content was measured between islets from donors with a BMI above and below 30 (Matsumoto et al. 2004; Lyon et al. 2016). Notably, none of our islet donors was severely obese: only 5/43 subjects had a BMI between 30 and 33. We confirmed a slight increase in islet size with BMI, but also measured an increase in islet insulin content after normalization for size (Fig. 6B). Since normalized insulin content does not increase with islet size only (Fig. 2D), there seems to exist a positive influence of body weight on islet insulin stores. One unprecedented finding of this study was the positive correlation between the amplitude of insulin secretion during stimulation with glucose, tolbutamide, or forskolin and BMI of the islets donor. The dynamics of the response was unaffected and the stimulation index only tended to increase. It appears, therefore, that the characteristic hyperactivity of *β*‐cells in obese subjects persists in vitro, at least when islets are tested within 3 days of isolation. An outstanding issue is whether this long‐lasting hyperactivity reflects an adaptation of all *β*‐cells or a recruitment of dormant *β*‐cells or islets (Pipeleers et al. 2017). Anyhow, matching for donor BMI is important in in vitro studies of islet function.

## Conclusions

The present analysis indicates that in vitro studies of insulin secretion using human islets within 1–3 days of isolation are unlikely to be confounded by many preparation characteristics or currently identified donor attributes. The most significant negative impact is produced by too low a purity of the preparations, which is easily avoidable. Conversely, insulin secretion is positively influenced by donor BMI and islet size, two parameters that should be matched in compared groups. Finally, it is important to emphasize that the present observations pertain to in vitro measurements of insulin secretion in normal islets and cannot necessarily be extrapolated to islets obtained from diabetic subjects, to studies of other islet features or to in vivo islet functioning after transplantation.

## Conflict of Interest

I have no conflict of interest to declare.
